# Roles of Hydrogen Sulfide Donors in Common Kidney Diseases

**DOI:** 10.3389/fphar.2020.564281

**Published:** 2020-11-19

**Authors:** Ebenezeri Erasto Ngowi, Muhammad Sarfraz, Attia Afzal, Nazeer Hussain Khan, Saadullah Khattak, Xin Zhang, Tao Li, Shao-Feng Duan, Xin-Ying Ji, Dong-Dong Wu

**Affiliations:** ^1^ School of Basic Medical Sciences, Henan University, Kaifeng, China; ^2^ Henan International Joint Laboratory for Nuclear Protein Regulation, Henan University, Kaifeng, China; ^3^ Department of Biological Sciences, Faculty of Science, Dar es Salaam University College of Education, Dar es Salaam, Tanzania; ^4^ Kaifeng Municipal Key Laboratory of Cell Signal Transduction, Henan Provincial Engineering Centre for Tumor Molecular Medicine, Henan University, Kaifeng, China; ^5^ Faculty of Pharmacy, The University of Lahore, Lahore, Pakistan; ^6^ College of Pharmacy, Henan University, Kaifeng, China; ^7^ Institute for Innovative Drug Design and Evaluation, School of Pharmacy, Henan University, Kaifeng, China; ^8^ Diseases and Bio-Safety, School of Basic Medical Sciences, Henan University, Kaifeng, China; ^9^ School of Stomatology, Henan University, Kaifeng, China

**Keywords:** H_2_S donors, physiological process, renal dysfunction, signaling pathways, common renal diseases

## Abstract

Hydrogen sulfide (H_2_S) plays a key role in the regulation of physiological processes in mammals. The decline in H_2_S level has been reported in numerous renal disorders. In animal models of renal disorders, treatment with H_2_S donors could restore H_2_S levels and improve renal functions. H_2_S donors suppress renal dysfunction by regulating autophagy, apoptosis, oxidative stress, and inflammation through multiple signaling pathways, such as TRL4/NLRP3, AMP-activated protein kinase/mammalian target of rapamycin, transforming growth factor-β1/Smad3, extracellular signal-regulated protein kinases 1/2, mitogen-activated protein kinase, and nuclear factor kappa B. In this review, we summarize recent developments in the effects of H_2_S donors on the treatment of common renal diseases, including acute/chronic kidney disease, renal fibrosis, unilateral ureteral obstruction, glomerulosclerosis, diabetic nephropathy, hyperhomocysteinemia, drug-induced nephrotoxicity, metal-induced nephrotoxicity, and urolithiasis. Novel H_2_S donors can be designed and applied in the treatment of common renal diseases.

## Introduction

The involvement of hydrogen sulfide (H_2_S) in a variety of health disorders has gained increased attention from researchers and scientists in recent years. This rotten egg-smelling, colorless, and poisonous gas is the third gasotransmitter to be associated with body homeostasis and disease progression. Others include carbon monoxide (Kaide et al., 2001) and nitric oxide (NO) (Wever et al., 1999). H_2_S levels have been established as a promising disease indicator and treatment for a number of health conditions including cancers (Szabo et al., 2013), neurodegenerative diseases (Vandini et al., 2019), cardiovascular diseases (Peter et al., 2013), osteoporosis (Ma et al., 2019), kidney diseases (Aminzadeh and Vaziri, 2011), obesity (Katsouda et al., 2018), and aging (Kasinath, 2018). The gas shows protective roles by mediating cellular activities such as autophagy (Ling et al., 2017), inflammation, apoptosis (Tan et al., 2015), and oxidative stress (Cheung and Lau, 2018). Tempering with H_2_S level exerts direct effects on several cellular pathways such as nuclear factor kappa B (NF-κB) (Du et al., 2019), transforming growth factor-β (TGF-β) (Huang et al., 2018), extracellular signal-regulated protein kinases 1/2 (ERK1/2) (Shi et al., 2019), AMP-activated protein kinase (AMPK), and the mammalian target of rapamycin (mTOR) (Wu et al., 2017).

H_2_S is synthesized both enzymatically and nonenzymatically in mammalian cells (Nagahara et al., 1998; Shibuya et al., 2009). Three enzymes, namely, cystathionine γ-lyase (CSE) (Chiku et al., 2009), cystathionine β-synthase (CBS) (Braunstein et al., 1971), and 3-mercaptopyruvate sulfurtransferase (3-MPST) (Shibuya et al., 2009), are involved in the production of H_2_S. CBS and CSE are specifically located in the cytoplasm, while 3-MPST is found in both cytoplasm and mitochondria (Nagahara et al., 1998; Banerjee et al., 2015). 3-MPST catalyzes the production of H_2_S from 3-mercaptopyruvate (3-MP), which is synthesized by cysteine aminotransferase (CAT) from cysteine (Cys) and α-ketoglutarate (Kuo et al., 1983; Shibuya et al., 2009). Meanwhile, CSE and CBS produce H_2_S by catalyzing homocysteine (Hcy) and Cys in the transsulfuration pathway (Chen et al., 2004; Chiku et al., 2009). Low production of H_2_S and downregulation of CBS, CSE, and 3-MPST expressions have been reported in common renal disorders (Aminzadeh and Vaziri, 2011; Han et al., 2017; Chen Q. et al., 2018; Chen Y. et al., 2018; Zhou et al., 2020). Although the mechanism in the reduction and underproduction of H_2_S in common renal diseases is not yet clear, the supplementation with H_2_S donors can effectively restore H_2_S levels and ameliorate the disease state associated with low production of H_2_S (Song et al., 2014; Weber et al., 2017; Chen et al., 2018; Khukhlina et al., 2018; Akbari et al., 2019). Hence, understanding of the mechanism of H_2_S production in the body at the normal physiological condition and low production in the disease state can be beneficial in renal disease management. Generally, the protective effects of H_2_S can be attributed to its interactions with cell receptors and channels (Zhao et al., 2001) and its roles in posttranslation modification of proteins via sulfuration/persulfidation (Sen et al., 2012) and phosphorylation (Cao et al., 2018).

Renal system is one of the key and complex body systems involved in the regulation of body fluid and electrolytes. Apart from its basic functions, the system is also a target for numerous disease conditions of different etiology ranging from inflammations, acute injuries to chronic injuries, and cancer. In 2017, a systematic analysis of the global burden of chronic kidney diseases (CKD) reveals that over 1.2 million deaths occurred worldwide due to CKD, which is an increase of more than 40% from 1990 (Bikbov et al., 2020). Renal diseases can be classified into primary or secondary renal diseases. Primary renal diseases are caused by less severe diseases such as glomerulonephritis and tubulointerstitial fibrosis that interferes the normal kidney functions, whereas secondary kidney diseases involve the diseases that affect the kidney as a result of the complication of their long-duration and poor management of diabetes type 2 mellitus, hypertension, and primary kidney diseases (Schiffl et al., 2002). The pathology of the kidney is categorized into four main anatomical subsets which include the diseases of the glomerulus (diabetes and neoplasia), interstitium (obstructions and urinary tract infections), tubules (renal tubular acidosis and cystinuria), and blood vessels (renal vascular diseases) (Levey and Coresh, 2012). The risk factors for renal diseases include smoking, aging, environmental pollutants, and diseases (such as obesity, diabetes, anemia, and hypertension). Their initiation and development involve several mechanisms such as inflammation, oxidative stress, apoptosis, matrix production, autophagy, and cell injury. H_2_S is involved in the regulation of these mechanisms, suggesting that H_2_S may have potential in the treatment of kidney diseases.

In this review, we will analyze recent developments on the roles of H_2_S donors in common renal disorders. In addition, we summarize H_2_S-associated cellular pathways to show possible target sites for these compounds.

## The Biosynthesis of Hydrogen Sulfide

### Major Sources of Hydrogen Sulfide in Human Body

**FIGURE 1:**
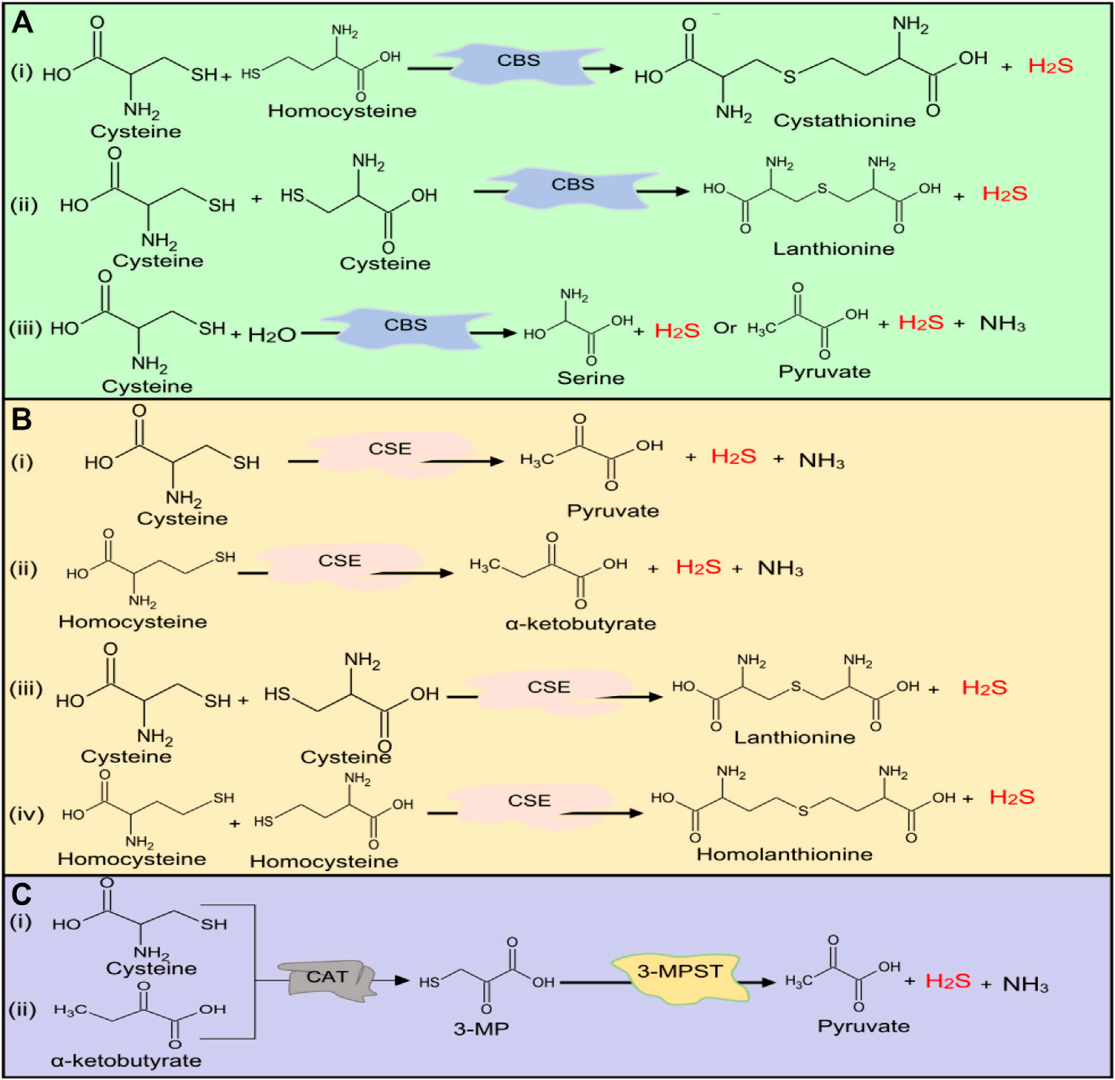
H_2_S production at normal physiological conditions in the body by **(A)** CBS, **(B)** CSE, and **(C)** 3-MPST. Both CBS and CSE produce H_2_S from cysteine and homocysteine, while 3-MPST produces H_2_S from 3-MP generated by CAT from Cys and α-ketobutyrate. H_2_S, hydrogen sulfide, CBS, cystathionine β-synthase; CSE, cystathionine γ-lyase; 3-MPST, 3-mercaptopyruvate sulfurtransferase; 3-MP, 3-mercaptopyruvate; CAT, cysteine aminotransferase; Cys, cysteine.

**FIGURE 2:**
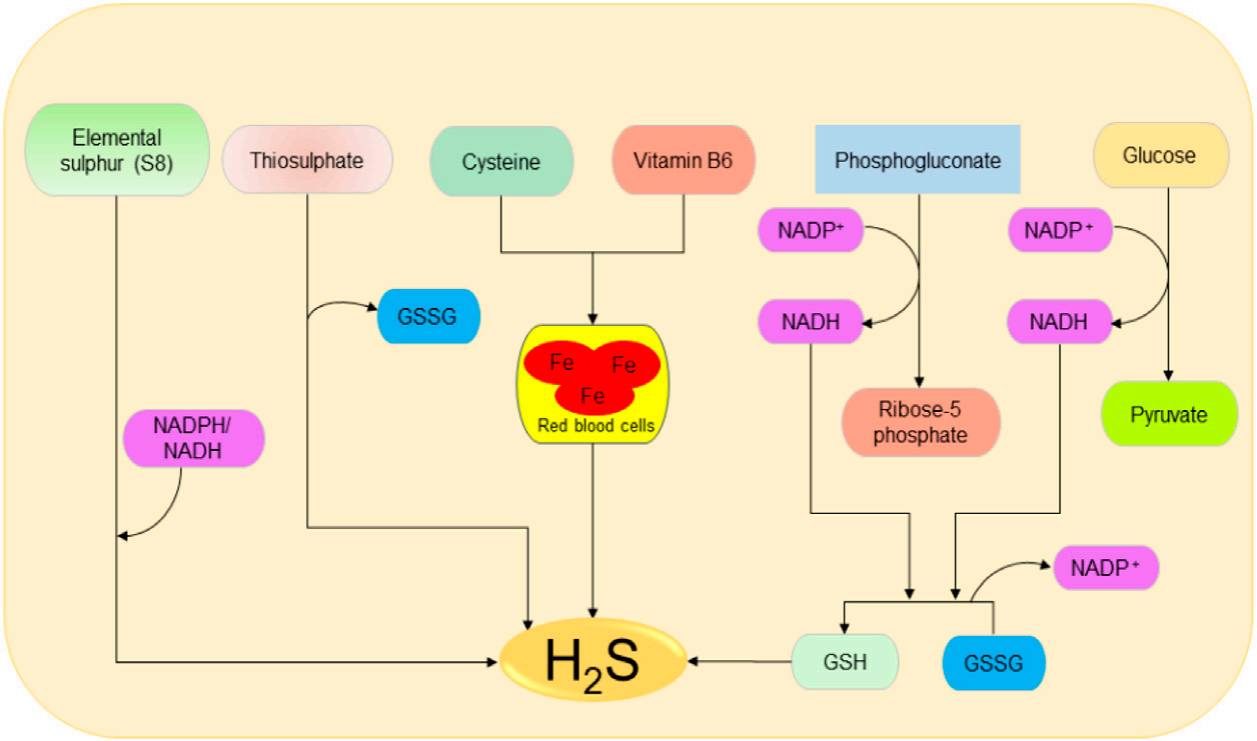
Possible spontaneous/nonenzymatic pathways involved in the production of H_2_S from elemental sulfur, thiosulfate, glutathione, cysteine, glucose, and phosphogluconate. Starting from the right-hand side, the two processes of glycolysis and NADPH oxidase facilitate the reduction of GSSG to GSH, which can be directly reduced to H_2_S. Next, in the presence of vitamin B6, cysteine can be catalyzed by iron contained in the red blood cells to produce H_2_S. Thiosulfate and elemental sulfur can also be reduced to H_2_S. H_2_S, hydrogen sulfide; NADPH, nicotinamide adenine dinucleotide phosphate oxidase; GSSG, oxidized glutathione; GSH, reduced glutathione.

The main source of H_2_S in the human body is Cys metabolism. Cys is nonessential sulfur, containing amino acid that contributes to the synthesis of other amino acids including glutathione (GSH) which is involved in regulation of oxidative stress and detoxification (Beatty and Reed, 1980; Baker and Czarnecki-Maulden, 1987; Sekhar et al., 2011). Besides, it also participates in the synthesis of nonprotein essential compounds such as taurine and coenzyme A (Ondarza et al., 1974; Penttilä, 1990). H_2_S is synthesized as a byproduct of the Cys metabolism. The enzymatic production of H_2_S involves two systems, including pyridoxal 5′-phosphate-dependent (CBS and CSE) and pyridoxal 5′-phosphate-independent (3-MPST) systems (Stipanuk and Beck, 1982; Mikami et al., 2011). CBS mediates the conversion of Hcy and serine to produce cystathionine, which is then converted by CSE into Cys, α-ketobutyrate, and ammonia (Stipanuk and Ueki, 2011). CBS facilitates β replacement of thiol (-SH) group of the Cys with Hcy to produce H_2_S; meanwhile, CSE facilitates α, β, and γ elimination of cystathionine, Hcy, and Cys to form H_2_S (Chiku et al., 2009). On the other hand, 3-MPST and CAT catalyze the production of H_2_S from cysteine and α-ketoglutarate (Shibuya et al., 2009). In addition, H_2_S can be produced nonenzymatically from several biological reactions involving Cys, thiosulfate, elemental sulfur, GSH, glucose, and phosphogluconate (Koj et al., 1967; Searcy and Lee, 1998; Kolluru et al., 2013; Yang et al., 2019). The enzymatic and nonenzymatic mechanisms involved in the production of H_2_S are summarized in Figures 1, 2, respectively.

### The Localization of Hydrogen Sulfide-Generating Enzymes in Kidney

CSE, CBS, and 3-MPST are localized in the kidney. In human renal tissues, CSE enzyme is reported to be localized in the glomerulus and tubulointerstitium (Bos et al., 2013). In addition, 75% of the renal cells and 87% of endothelial cells express the enzymes. On the other hand, CBS is highly expressed in renal proximal tubular cells (Yuan et al., 2017). Similarly, in mice kidneys, CBS and CSE proteins are well expressed with the former reported to be predominantly in the outer renal cortex (mainly in the proximal convoluted tubule) and the later in the inner cortex and medulla (specifically in the proximal straight tubule) (House et al., 1997). In addition, 3-MPST is localized in the proximal tubular epithelium (Nagahara et al., 1998). The deficiency of H_2_S-producing enzymes and H_2_S levels has been reported in human patients and rat models, which can be correlated with the severity of kidney diseases (Yuan et al., 2017). The above evidence indicates that the kidneys are enriched with all three enzymes and confirm their potential roles in kidney function.

## The Role of Hydrogen Sulfide in Kidney Physiology

### Glomerular Filtration Rate

**FIGURE 3:**
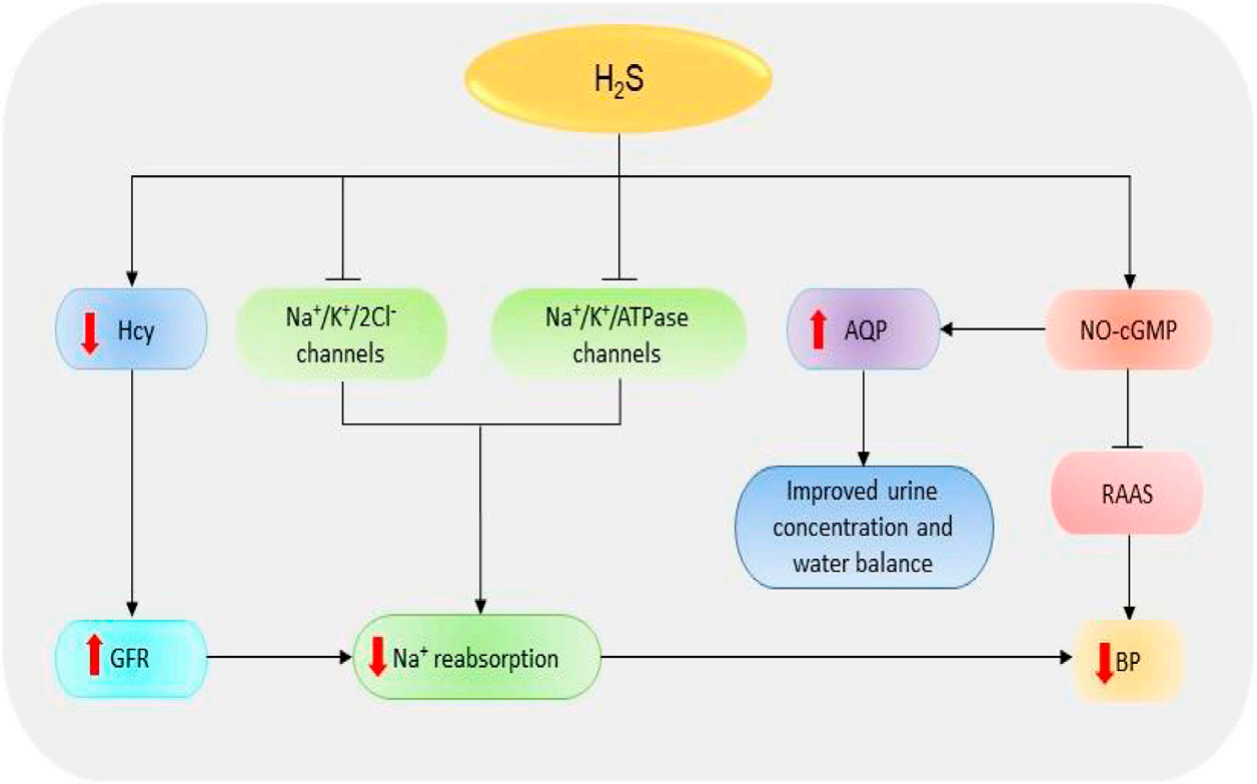
The dramatic presentation of the kidney functions regulated by H_2_S. H_2_S stimulates NO-cGMP pathway resulting in an inhibition of RAAS and subsequent reduction in BP. The activation of NO-cGMP can also increase the expressions of AQP-2, thereby improving urine concentration and water balance. Besides, H_2_S induces the inhibition of Na^+^/K^+^/2Cl^-^ and Na^+^/K^+^/atpase channels, thence reducing Na^+^ reabsorption and consequently decreasing the BP. H_2_S also reduces Hcy levels resulting in an increase in GFR, suppression of Na^+^, and finally decline in BP. H_2_S, hydrogen sulfide; Hcy, homocysteine; Na^+^, sodium; NO, nitric oxide; cGMP, cyclic guanosine monophosphate; GFR, glomerular filtrate rate; BP, blood pressure; AQP-2, aquaporin-2; RAAS, renin-angiotensin-aldosterone system.

Glomeruli are the basic units of the kidney. They are tiny filters that play crucial roles in the removal of waste material and urine formation (Pollak et al., 2014). Low glomerular filtration rate (GFR) has been reported in several renal diseases including acute kidney injury (AKI) and CKD (Marsik et al., 2008; Hsu and Hsu, 2011). Decreased GFR affects the removal of waste products by the kidneys. In a recent study, the reduced plasma levels of H_2_S in CKD patients could be significantly correlated with the reduction in GFR which confirms the importance of the gas in regulating the activities of the glomerulus (Kuang et al., 2018). It has been revealed that treatment of anesthetized rat models with CBS and CSE inhibitors, aminooxyacetic acid, and DL-propargylglycine (PAG) respectively, can impede the GFR by reducing vasodilation of the preglomerular arterioles; however, treatment with low doses of NaHS (a fast H_2_S-releasing donor) can protect the glomerulus functions by inducing opposite effects (Xia et al., 2009). Thus, treatment with H_2_S donors can improve kidney condition in patients by improving GFR (Figure 3).

### Sodium Reabsorption

The reabsorption of sodium (Na^+^) is one of the primary functions of the renal system. The failure of the kidney to remove excess Na^+^ has detrimental pathological implications due to its involvement in the regulation of blood volume, blood pressure (BP), and fluid balance. In addition, the accumulation of Na^+^ facilitates kidney damage by increasing fibrosis and oxidative stress (Thijssen et al., 2008). NaHS treatment can decrease Na^+^ reabsorption through several mechanisms including the elevation of GFR and the inhibition of Na^+^/K^+^/2Cl^-^ and Na^+^/K^+^/ATPase channels, leading to the high urinary Na^+^ concentration (Xia et al., 2009). It has also been shown that treatment with H_2_S donor NaHS prevents the activation of epithelial Na^+^ channels by regulating the reactive oxygen species (ROS)/phosphatidylinositol 3-kinase/phosphatase and tensin homolog signaling pathway (Zhang et al., 2013; Wang et al., 2015). Together, these data imply that H_2_S is involved in Na^+^ balance and the regulation of kidney functions.

### Blood Pressure Regulation

Another key function of the kidney is the regulation of BP. Kidney works together with the renin-angiotensin-aldosterone system (RAAS) to regulate BP and maintain electrolyte balance (Cortinovis et al., 2016). The decline in BP and Na^+^ level or the stimulation of β1-adrenoreceptors can result in the secretion of renin, which in turn converts angiotensinogen (produced in the liver) into angiotensin-I which is then converted to angiotensin II by angiotensin-I converting enzyme (ACE) (Soubrier et al., 1993; Deschepper, 1994). Angiotensin II acts on several receptors to mediate the downstream effects. For instance, angiotensin II facilitates the secretion of aldosterone hormone which is involved in the elevation of BP by promoting the reabsorption Na^+^ and excretion of potassium via Na^+^/K^+^/ATPase channels (Ikeda et al., 1991; Giacchetti et al., 1996). Treatment with a phosphorothioate-based synthetic H_2_S donor JK-1 suppresses the activation of RAAS, reduces BP, and improves renal functions (Li et al., 2018). It has also been shown that the treatment of anesthetized rat models with H_2_S donor Na_2_S can significantly decrease the BP and mesenteric resistance; meanwhile, the administration of CSE inhibitors (β-cyanoalanine) and PAG could increase BP and resistance in both mesenteric and renal circulations (Morales-Loredo et al., 2019). Another component involved in BP regulation is cyclic guanosine monophosphate (cGMP). The NO-cGMP pathway participates in the regulation of BP by attenuating the expression of kidney renin levels and the synthesis of aldosterone, ACE, and angiotensin II-type I receptor, resulting in the downstream reduction of BP (Shesely et al., 1996; Takemoto et al., 1997; Usui et al., 1998; Gambaryan et al., 2003). A recent report indicates that treatment with NaHS can also facilitate the release of NO and prevent cGMP breakdown by inactivating phosphodiesterase type 5, thereby maintaining the activation of NO-cGMP pathway (Coletta et al., 2012). In contrast, CSE inhibition inactivates the pathway and the downstream responses. Further evidence indicates that cGMP is downregulated in CSE knock-out mice and that the administration of NaHS results in its stimulation and the subsequent activation of protein kinase G (PKG), thus leading to vasodilation (Bucci et al., 2012). In addition, H_2_S can mediate nonclassical cGMP-independent activation of PKG via antioxidants to induce BP regulation (Stubbert et al., 2014). It has also been reported that PKG-inhibitor could not reduce GYY4137 (a slow H_2_S-releasing donor)-induced vasodilation of mouse aorta which suggests that the donor signals via PKG-independent pathway (Bucci et al., 2012). The data suggest that H_2_S regulates BP by stimulating both cGMP-dependent/independent and PKG-independent pathways.

### Water Balance and Urine Formation

Through its involvement in urine formation, the kidney actively participates in water regulation. Its role in water balance is supported by high expressions of water regulating proteins known as aquaporin (AQP) (Ishibashi et al., 2009). AQP-2 has been reported to be downregulated in animal models of kidney disorders including AKI and unilateral ureteral occlusion (UUO) (Suh et al., 2015; Wang et al., 2015). A recent animal study demonstrates that administration of H_2_S inhibitors, aminooxyacetic acid and PAG, can cause urinary concentration defects and reduce the expressions of AQP-2; however, treatment with GYY4137 and NaHS improves the urine concentration and upregulates AQP-2 proteins possibly via cAMP-dependent protein kinase signaling pathway (Luo et al., 2019). This implies that H_2_S plays a vital role in water balance and urine formation; however, further studies are needed to illuminate the mechanism involved.

## The Mechanism of Action of Hydrogen Sulfide in Common Kidney Diseases

### Oxidative Stress

**FIGURE 4:**
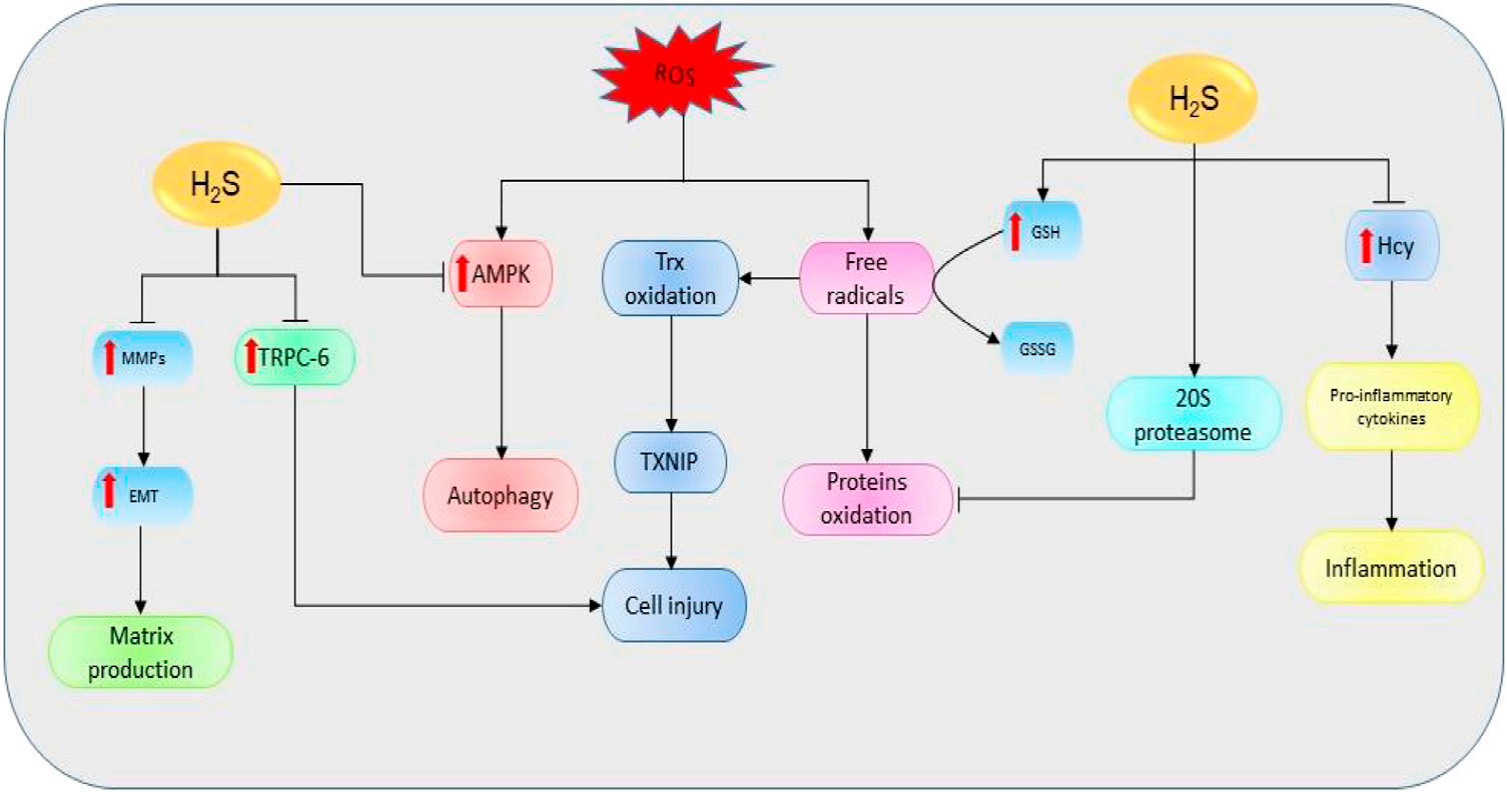
The diagrammatic illustration of the influence of H_2_S on the mechanisms involved in the initiation and development of renal diseases. H_2_S suppresses the elevation of Hcy resulting in the downregulation of proinflammatory cytokines (such as IL-1β, IL-6, and NF-қB) and the corresponding reduction of proinflammatory activities. Moreover, H_2_S increases the expressions of 20S proteasome which then reduces the oxidation of proteins by mediating proteolytic degradation of the oxidized proteins. Next, H_2_S can also stimulate the oxidation of GSH to GSSG resulting in the reduction of free oxygen radicals and subsequent suppression of protein oxidation. By reducing free oxygen radicals level, H_2_S also prevents the oxidations of Trx and its binding into TXNIP resulting in reduction of cell injury. Next, H_2_S prevents the ROS-mediated activation of AMPK pathway and its downstream effect on the induction of autophagy. Similarly, H_2_S reduces the expressions of MMPs and their resulting effects in EMT and matrix production. Also, H_2_S suppresses the levels of TRPC-6 leading to a reduction in cell injury. Alternatively, the decline in H_2_S levels could reverse the protective effects, resulting in the promotion of mechanisms favoring the development of kidney diseases (H_2_S, hydrogen sulfide; Hcy, homocysteine; GSH, glutathione; GSSG, oxidized glutathione; ROS, reactive oxygen species; TRPC-6, transient receptor potential cation channel, subfamily C, member 6; MMPs, matrix metalloproteinases; EMT, epithelial-mesenchymal transition; Trx, thioredoxin; TXNIP, Trx-interacting protein; AMPK, AMP-activated protein kinase).

Oxidative stress simply refers to the cellular redox imbalance in favor of oxidation which leads to the formation of radicals (Sies, 1997). Defects in antioxidant status result in higher levels of free oxygen radicals and superoxide dismutase, GSH peroxidase, and lipid peroxide have been identified as potential indicators for AKI (Dubey et al., 2000). These radicals cause DNA, protein, and lipid oxidations, thereby affecting their structure and functions. Evidence shows that the inhibition of CSE in mice models is critical for the production ROS and mitochondria dysfunction (Han et al., 2017). However, the elevation of H_2_S levels can restore the functions and reduce oxidative levels (Shan et al., 1990). One of the main mechanisms used to destroy unfolded and oxidized proteins is an adenosine triphosphate-independent 20S proteasome of the ubiquitin proteolytic pathway (Davies, 2001). It has been revealed that the inhibition of 20S proteasome promotes kidney malfunctioning, therefore exacerbating the progression of AKI and renal ischemia/reperfusion (I/R) (Huber et al., 2009; Zhang et al., 2020). However, NaHS could improve kidney condition by elevating the levels of proteasome subunit alpha type 6 and proteasome subunit beta type 7 (the key subunits of 20S proteasome), thereby reducing endoplasmic reticulum (ER) stress in rat models (Yi et al., 2018). In summary, H_2_S can regulate kidney activities via its interaction with ROS and 20S ubiquitin degradation pathway (Figure 4).

### Inflammation

Inflammation is one of the key players in kidney pathology. In patients with interstitial fibrosis and end-stage renal disease (ESRD), the plasma levels of inflammatory-mediated receptors and tumor necrosis factor receptor-1 and -2 are highly elevated, and the event is associated with the severity of the diseases (Niewczas et al., 2012; Sonoda et al., 2015). Moreover, the serum levels of several proinflammatory cytokines including interleukin (IL)-1β, IL-6, IL-8, and tumor necrosis factor-α (TNF-α) are also reported to be increased (Goldstein et al., 2006). With respect to H_2_S, the expression of CBS is reduced in renal tissues of patients with obstructive nephropathy compared to the normal ones (Yuan et al., 2017). Further analysis reveals that the knockdown of CBS in HK-2 human proximal tubular derived cells results in the elevation of proinflammatory markers while its overexpression shows opposite effects (Yuan et al., 2017). Moreover, both CSE and CBS negatively regulate Hcy levels via transsulfuration pathway; hence, their deficiency can cause the elevation of Hcy and the corresponding activation of proinflammatory responses leading to the progression of fibrosis (Chiku et al., 2009; Shastry and James, 2009; Yang et al., 2016). These results indicate that the reduction of the deficiency of H_2_S-producing enzymes in kidney diseases corresponds to the accumulation of Hcy and associated inflammatory features; thence, its supplementation could be useful in treating these diseases.

### Autophagy

Autophagy is an important process associated with the destruction of unwanted cellular components and nutrients recycling. It plays a key role in protecting renal cells from apoptosis and associated loss of functions (Jiang et al., 2012). The deletion of autophagy-related gene 5 in mice results in the accumulation of oxidized and unfolded proteins, proteinuria, and subsequently podocyte loss (Hartleben et al., 2010). Besides, autophagy plays a crucial role in maintaining autosomal biogenesis and kidney functions and its loss could lead to the promotion of kidney damage (Maejima et al., 2013). However, further evidence suggests that excessive autophagy can stimulate apoptotic activities, leading to renal tubular atrophy (Chen et al., 2018). In UUO-induced mice, CBS and CSE levels have been shown to be reduced; meanwhile, the levels of autophagy and apoptotic markers are considerably elevated (Li et al., 2010). Treatment with exogenous H_2_S could protect the kidneys from ROS-induced autophagy and renal damage by inhibiting the ROS-AMPK pathway (Chen et al., 2018). In brief, the data above suggest that the decline in H_2_S levels in renal diseases can enhance autophagy activities through the elevation of ROS levels, resulting in disease progression; however, supplementation of H_2_S can rescue the condition.

### Matrix Production

Extracellular matrix (ECM) accumulation drives the loss of renal function and progression of renal damage. The activation of transforming growth factor-β1 (TGF-β1) is highly associated with the advancement of kidney fibrosis through its influence on the deposition of ECM (Yamamoto et al., 1994). Mechanistically, TGF-β1 increases the expressions of matrix metalloproteinases (MMPs) including MMP-2 and MMP-9, resulting in the promotion of epithelial-mesenchymal transition (EMT) and associated profibrotic activities (Turck et al., 1996; Strutz et al., 2002; Zhao et al., 2013). It has been suggested that the downregulation of CSE and CBS can induce matrix remodeling by negatively regulating the expressions of MMP-9, connexins, and N-methyl D-aspartate receptors in mice (Kundu et al., 2013). However, the effect could be ameliorated with NaHS treatment (Kundu et al., 2015). Furthermore, the treatment of diabetic mice model with tadalafil (a phosphodiesterase 5 inhibitor and H_2_S donor) reduces matrix production in podocytes by regulating NO-H_2_S-AMPK-mTORC1 signaling cascades (Lee et al., 2015). Treatment with GYY4137 has also been indicated to stabilize the levels of MMP-9, MMP-13, and MMP-14 and reduce ROS-induced kidney fibrosis by elevating the level of microRNA-194 (John et al., 2017). In summary, the decline in H_2_S levels facilitates the production of a matrix by regulating the associated pathways; however, the upregulation of H_2_S can prevent the event and improve kidney functions.

### Cell Injury

Cell injury affects the tissue repair process by inducing further infiltrations and loss of function. One of the main mediators of cell injury is hypoxia-induced mitochondria dysfunction (Zhang et al., 2007; Su et al., 2013; Zhu et al., 2014). Hypoxia-inducible factors (HIFs) play essential roles in kidney cell repair by reducing apoptosis, promoting cell proliferation, and facilitating the release of specific genes associated with tissue repair (Zhang et al., 2003). Although primary injuries can be well repaired, the defects in the repair mechanism can augment the condition, thus resulting in tissue and organ injury. For instance, HIF activation can prevent the degeneration of tissue by improving oxygen balance and promoting cell repair (Bernhardt et al., 2006); however, its prolonged stimulation can result in the progression of tissue injury by increasing ECM accumulation and inflammatory responses (Haase, 2012; Conde et al., 2017). The injuries are the result of high apoptosis, podocyte loss, accumulation of ECM, and inflammation. Besides, another study shows that immortalized proximal tubule cells release kidney injury molecule-1 which stimulates the secretion of monocyte chemoattractant protein-1 and its macrophage-dependent activities thereby promoting kidney fibrosis (Humphreys et al., 2013). A previous report indicates that the downregulation of CSE enhances Hcy-mediated podocyte injury by regulating Wnt pathway (Liu et al., 2015). Moreover, the inhibition of CSE promotes cell injury by facilitating the oxidation of thioredoxin (Trx) and its binding into Trx-interacting protein, resulting in the inhibition of apoptosis signal-regulating kinase 1 and its effect on P38 mitogen-activated protein kinase (MAPK) pathway; however, treatment with NaHS could reverse the effects (Mao et al., 2019). It has also been shown that the treatment of diabetic kidney disease mice model with taurine (a H_2_S-releasing sulfur-containing amino acids) significantly elevates the CSE level, thereby increasing H_2_S production and downregulating the protein levels of podocyte homeostasis regulator, transient receptor potential cation channel, subfamily C, member 6, and consequently the reduction of glucose-induced podocytes injuries (Zhang et al., 2020). Taken together, the above information implies that the downregulation of H_2_S-producing enzymes in kidney diseases could cause cell injury; however, the problem can be rescued with supplementation of H_2_S.

## The Role of Hydrogen Sulfide in Common Kidney Diseases

### Acute Kidney Injury

**FIGURE 5:**
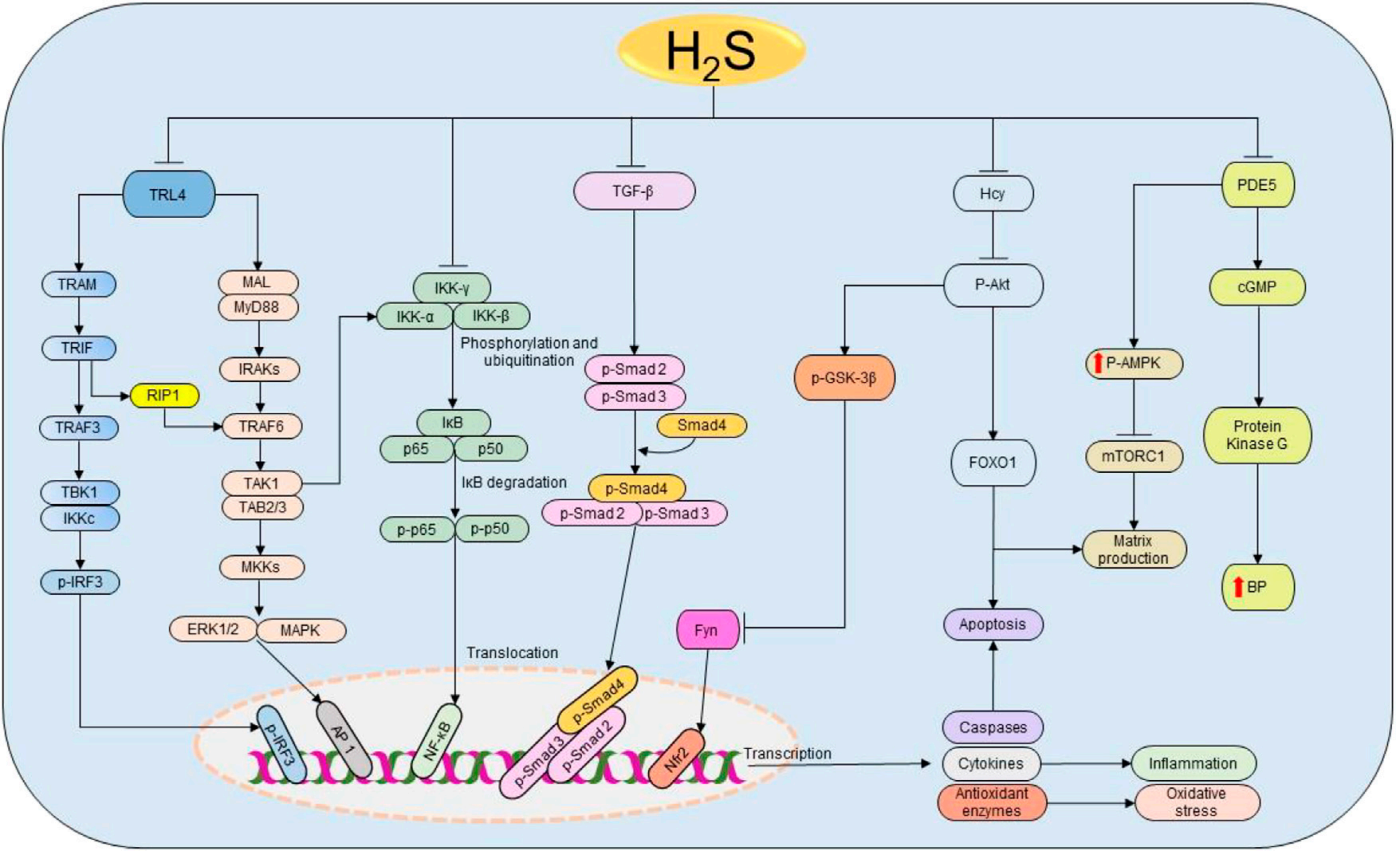
The cellular pathways regulated by H_2_S in renal diseases. Starting from the right-hand side, H_2_S suppresses the expressions of PDE5, thereby enhancing the production of NO and the activation of cGMP and PKG resulting in the subsequent reduction of BP. Simultaneously, PDE5 inhibition facilitates the phosphorylation of AMPK, resulting in the inactivation of mTORC1 and the corresponding reduction of matrix production. Next, H_2_S reduces Hcy levels, thereby inducing the dephosphorylation of Akt and the downstream activation of FOXO 1 signaling pathway, leading to the reduction of apoptosis and matrix production. The deactivation of Akt also triggers the activation of GSK3β and inhibition of Fyn leading to the elevation on Nfr2 and consequent rise in the antioxidant status. Similarly, H_2_S attenuates the activation of TGF-β and its downstream effectors Smad2/3 resulting in the promotion of anti-inflammatory activities. Moreover, H_2_S prevents the phosphorylation and ubiquitination of IKK complex, thereby facilitating the inhibition of NF-κB and subsequently reducing inflammation. H_2_S also inhibits TRL4-induced activation of ERK1/2, p38 MAPK, and NLRP3 pathways resulting in the suppression of apoptosis, oxidative stress, and inflammation. H_2_S, hydrogen sulfide; PDE5, phosphodiesterase type 5; cGMP, cyclic guanosine monophosphate; BP, blood pressure; p-AMPK, phosphorylated AMP-activated protein kinase; mTORC1, the mammalian target of rapamycin complex 1; FOXO1, forkhead box protein O1; Akt, protein kinase B; GSK3β, glycogen synthase kinase β; TGF-ꞵ1, transforming growth factor-beta 1; Smad 2/3/4, mothers against decapentaplegic homolog 2/3/4; IKK, IκB kinase; IκB, inhibitor of nuclear factor kappa B; p50 and p65, nuclear factor kappa B subunits; NF-κB, nuclear factor kappa B; TRL4, Toll-like receptor 4; MyD88, myeloid differentiation primary response 88; MAL, MyD88 adapter-like proteins; IRAK, interleukin-1 receptor-associated kinases; TRAF, tumor necrosis factor receptor-associated factor; TAK1, TGF-α activating kinase 1; TAB 2/3, TAK 1 binding protein 2/3; MKK, MAPK kinases; TRAM, translocating chain-associating membrane protein; TRIF, TIR-domain-containing adapter-inducing interferon β; RIP1, receptor-interacting serine/threonine-protein kinase 1; TBK1, TANK-binding kinase 1; IRF3, interferon regulatory factor 3; ERK1/2, extracellular signal-regulated kinase 1/2; MAPK, mitogen-activated protein kinase; AP-1, activator protein 1; NLPR3, NOD-, LRR-, and pyrin domain-containing protein 3.

AKI is the condition caused by the drastic rise in urine levels of creatinine and urea as a result of decreased GFR (Hilton, 2006). The causes include infections, sepsis, liver failure, heart failure, cancer, kidney stones, I/R, glomerulonephritis, and medications. Oxidative stress and inflammation have been indicated as the main targets for H_2_S in kidney injury treatment induced by sepsis and renal I/R (Bos et al., 2009; Chiku et al., 2009; Wu et al., 2017; Qiu et al., 2018). Sepsis-associated AKI patients have high levels of creatinine, urea nitrogen, IL-1β, TNF-α, myeloperoxidase, and malondialdehyde concentrations, and these features are significantly associated with low H_2_S levels (Chen et al., 2018). In addition, treatment with NaHS effectively attenuates inflammation and oxidative activities by regulating Toll-like receptor 4/NOD-, LRR-, and pyrin domain-containing protein 3 (NLRP3) pathway in lipopolysaccharide-induced AKI mice model (Chen et al., 2018) (Figure 5). Considering the above findings, regardless of the causes, the protective effect of H_2_S donors in AKI is conserved, which confirms the key role played by H_2_S in kidney injury and its therapeutic potential.

### Chronic Kidney Diseases

CKD is one of the common health diseases with more than 675 million cases and 1.2 million deaths recorded in 2017 (Bikbov et al., 2020). The term CKD covers all five stages of the gradual loss of kidney functions. The higher stages (3–5) are usually referred to as chronic renal failure (CRF) (Padmanabhan et al., 2017). Based on the estimated glomerulus filtration rate (eGFR), CKD stages are classified as follows: eGFR, stage 5 (eGFR ≤14), stage 4 (15 ≤ eGFR ≤29), stage 3 (30 ≤ eGFR ≤59), stage 2 (60 ≤ eGFR ≤89), and stage 1 (eGFR ≥90) (Yassine et al., 2016). CKD mainly results from mild-to-severe range of prerenal, intrinsic renal, and/or postrenal processed diseases. Reduced level of H_2_S has been reported in both CKD patients and experimental models (Aminzadeh and Vaziri, 2011; Kuang et al., 2018). In 5/6 nephrectomy mice model, NaHS could effectively improve kidney functions by promoting antioxidant, antiapoptotic, and anti-inflammatory responses via the activation and deactivation of nuclear factor erythroid 2-related factor 2 (Nfr2) and mTOR pathways, respectively (Shirazi et al., 2019). Moreover, treatment with 30 *µ*M NaHS for 8 weeks significantly decreases the expressions of cleaved caspase-3, p-NF-κB, and urine concentrations of neutrophil gelatinase-associated lipocalin in 5/6 nephrectomy mice (Askari et al., 2018). A recent study indicates that NaHS mitigates the protein levels of proinflammatory and proapoptotic markers in adenine-induced CRF in mice model by regulating MAPK and NF-κB pathways (Wu et al., 2017). Collectively, low level of H_2_S contributes to CKD progression by promoting apoptosis, autophagy, inflammation, and oxidative stress, and increasing the level of H_2_S could prevent these events, suggesting that H_2_S may serve as a new treatment option for CKD.

### Renal Fibrosis

Renal fibrosis is regarded as the hallmark for all progressive CKD (Zhou and Liu, 2016). The causes for the disease include diabetes, obstructive urinary, glomerulonephritis, glomerulosclerosis, hypertrophy, and mutation (Nogueira et al., 2017). Several factors such as autophagy, inflammation, accumulation of collagen, oxidative stress, and apoptosis are known to play roles in the progression of the disease. H_2_S treatment reverses the streptozotocin (STZ)-induced accumulation of collagen II, tissue inhibitor of metalloproteinases 2, kidney hydroxyproline, and downregulation of connexins and MMP1/2 in mice (Zeng et al., 2016). Furthermore, NaHS reduces the elevated apoptotic and inflammatory activities in STZ diabetic rats by regulating TGF-β1 and ERK1/2 signaling pathways (Li et al., 2017). A recent study shows that NaHS administration in diabetic mice restores the levels of serum creatinine, blood urea nitrogen, and inflammatory cytokines as well as inhibiting the activation of TGF-β1/Smad 3 pathway (Jia et al., 2018). The data indicate a potential link between H_2_S and ECM remodeling activities, through which it suppresses renal fibrosis.

### Unilateral Ureteral Occlusion

Obstructive uropathy is a renal disorder characterized by the disruption of the normal tubular fluid/urine flow from the kidney to the bladder as a result of structural/functional defects in the urinary tract (Klahr, 1991; Klahr, 2000). UUO is the common form of obstructive uropathy and an outstanding prototype for investigating tubulointerstitial fibrosis and obstructive nephropathy leading to CKD (Vaughan Jr et al., 2004). High inflammatory response and interstitial macrophage infiltration are associated with the induction of UUO (Silverstein et al., 2003). Growing evidence indicates that UUO diminishes the production of H_2_S and the expressions of CSE, CBS, and 3-MPST proteins (Han et al., 2017; Chen Q. et al., 2018; Chen Y. et al., 2018; Zhou et al., 2020). The deletion of CSE gene intensifies UUO-induced kidney fibrosis by increasing the interstitial collagen deposition, microphages infiltration, proinflammatory cytokine TNF-α and neutrophil marker Ly6G, mitochondrial damage, oxidative stress, and apoptosis (Han et al., 2017). However, the administration of H_2_S donors could restore UUO-induced alterations of the renal functions (Jiang et al., 2014). For instance, NaHS reverses the UUO-induced changes by suppressing the corresponding pathways, namely, NLRP3 (Chen et al., 2018), TGF-β1 (Jung et al., 2013; Song et al., 2014), and ROS-AMPK signaling cascades (Zhou et al., 2020). Likewise, GYY4137 can significantly reduce renal fibrosis, inflammation, and apoptosis in UUO-induced male Lewis rats by reducing the levels of EMT markers and inhibiting TGF-β1/Smad and MAPK pathways (Lin et al., 2018). In summary, the induction of UUO decreases the production of H_2_S and the inhibition of H_2_S-producing genes in UUO model could exacerbate the condition. However, the situation can be intervened and restored with the administration of H_2_S donors. Therefore, H_2_S donors may serve as a novel therapeutic option for the treatment of renal diseases associated with UUO.

### Focal Segmental Glomerulosclerosis

Focal segmental glomerulosclerosis (FSGS) is the glomerular disease caused by the formation of scars in the glomeruli and podocyte loss (Wharram et al., 2005; Woroniecki and Kopp, 2007). In an attempt to compensate for the loss, the remaining podocytes undergo hypertrophy (Wiggins et al., 2005); however, the insufficiency of this process to fulfill the increased demand of the glomerulus volume leads to the progression of FSGS (Kriz, 2012). Glomerular extracts from FSGS patients’ biopsy show elevated levels of mTOR and parietal epithelial cells (PEC)-activation associated genes (Puelles et al., 2019). In animal models, prolonged activation of mTOR pathway is positively correlated with PEC activation and FSGS progression (Zschiedrich et al., 2017; Puelles et al., 2019). Furthermore, the complete inhibition of the pathway aggravates the condition; meanwhile, partial inhibition restores glomerulus integrity and maintains podocyte hypertrophy (Zschiedrich et al., 2017). Treatment of hyperhomocysteinemia (HHcy)-induced mice with NaHS prevents glomerulosclerosis by improving GFR and reducing macrophage infiltration and proinflammatory activities (Sen et al., 2009; Sen et al., 2010). Another study indicates that NaHS can alleviate glomerulosclerosis symptoms in aging mice mainly by reducing urinary albumin, serum cystatin c, proinflammatories, and renal cortical levels of laminin γ1 (a matrix protein involved in glomerulosclerosis) and by activating AMPK pathway (Lee et al., 2018). In summary, H_2_S supplementation decreases matrix deposition in glomerulosclerosis by regulating AMPK-mTOR signaling cascades; however, further studies are needed to elucidate the mechanism involved.

### Hyperhomocysteinemia

HHcy refers to the elevated plasma level of Hcy usually above 15 µM (Seshadri et al., 2002). High level of HHcy is considered as a risk factor for various diseases including end-stage renal disease and neurodegenerative disorders (Seshadri et al., 2002; Nair et al., 2005). HHcy mice show reduced levels of CSE and CBS; conversely increasing H_2_S level decreases Hcy levels (Sen et al., 2010). HHcy promotes the dephosphorylation of Akt and FOXO1 signaling cascades, resulting in the subsequent activation of FOXO1 (Zhang et al., 2005), a vital pathway involved in apoptosis, oxidative stress, and inflammation (Ponugoti et al., 2013; Shi et al., 2018). Treatment with GYY4137 downregulates proapoptotic caspases, MMPs, collagens, and fibronectin one in HHcy-treated mouse mesangial cells through the regulation of Akt-FOXO1 pathways (Majumder et al., 2019). Similarly, GYY4137 reduces the expressions of caveolin 1 and connexins and increases the activities of endothelial NO synthase (eNOS) and tissue inhibitors of metalloproteinases (TIMPs) in HHcy mice (John and Sen, 2019). Furthermore, the treatment of HHcy mice with 30 *µ*M NaHS protects renal tissues from HHcy-induced inflammation and ROS by restoring MMPs/TIMPs and oxidized GSH/GSH status (Sen et al., 2009). NaHS also reduces the homocysteinylation of eNOS and stabilizes systolic BP, GFR, and connexins levels in HHcy mice (Pushpakumar et al., 2019). Based on the above information, there is a strong link between H_2_S and Hcy levels. Therefore, H_2_S supplementation could be a potential therapeutic option for treating HHcy-associated diseases.

### Diabetic Nephropathy

Diabetic nephropathy (DN) is a condition characterized by the presence of abnormal levels of protein in urine (proteinuria) (Østerby et al., 1993; Parving, 2001). DN is considered as an independent risk factor for ESRD (Ruggenenti et al., 1998). Reduced levels of H_2_S have been extensively documented in DN patients and mouse model (Yamamoto et al., 2013; Li et al., 2014). A recent study indicates that treatment with NaHS restores behavioral and biochemical changes in STZ-induced diabetic mice, while pretreatment with PAG (a CSE inhibitor) prevents the protective effects of NaHS (Kaur et al., 2015), which confirms the crucial role of H_2_S in the development and progression of DN. In addition, NaHS improves the GFR and blood flow by reducing angiotensin II level and regulating NF-κB, MAPK, and Nfr2 pathways (Zhou et al., 2014). Similarly, treatment with S-propargyl-cysteine improves inflammatory responses, oxidant/antioxidant status by inhibiting the activation of TGF β1/Smad3 and the phosphorylation of ERK, p38, and signal transducers and activators of transduction-3 signaling cascades in STZ-induced diabetic mice (Qian et al., 2016).

The overexpression of energy-sensing protein sirtuin 1 (SIRT1) impedes the progression of DN and reduces podocyte loss and oxidative stress in DN mice (Hong et al., 2018). SIRT1 regulates cellular activities by deacetylating several transcription factors including p53, p65, and FOXO (Solomon et al., 2006; Hariharan et al., 2010; Yang et al., 2012). Administration of 100 *μ*mol/kg/day NaHS protects renal cells from diabetes mellitus-induced damage by upregulating SIRT1 (Ahmed et al., 2019). Overall, H_2_S donors can prevent the progression of DN by regulating the associated cellular pathways, indicating that these donors could be a potential option for the treatment of DN.

### Drug-Induced Nephrotoxicity

The pathological effects of drugs are the main cause of hospital-acquired AKI (Kaufman et al., 1991). Some of these drugs include acetaminophen (APAP), aspirin, cisplatin, and indinavir (Naughton, 2008). Cisplatin, an anticancer drug, induces nephrotoxicity notably through the reaction involving platinum and thiol protein groups (Levi et al., 1980). One of the most investigated reactions is that of cisplatin and GSH (Dedon and Borch, 1987; Jansen et al., 2002). H_2_S protects the kidney from cisplatin-induced nephrotoxicity by increasing GSH level and regulating key cellular activities such as oxidative stress, inflammation, and apoptosis (Dwivedi et al., 1996; Chiarandini et al., 2008; Ahangarpour et al., 2014; Liu et al., 2016). A previous study indicates that pretreatment with 21 mg/kg/day GYY4137 for 3 days followed by the administration of cisplatin aggravates the renovascular damage by promoting apoptotic, oxidative stress, and inflammatory activities (Liu et al., 2016). Despite the problem observed in the methodology of this study, pretreatment with GYY4137 could not prevent the cytotoxicity of cisplatin. Alternatively, it has been reported that treatment with GYY4137 and NaHS exert antitoxicity effects through the reduction of ROS and the inhibition of nicotinamide adenine dinucleotide phosphate and MAPK activities in cisplatin-administered porcine renal proximal tubule cell line LLC-PK1 (Cao et al., 2018). NaHS can also upregulate the expression of nephrin and reduce the level of desmin (Karimi and Absalan, 2017). In addition, treatment with diallyl disulfide (DADS) alleviates nephrotoxic effects of cisplatin by increasing antioxidant defense and suppressing apoptotic, oxidative, and inflammatory activities (Elkhoely and Kamel, 2018). Collectively, treatment with H_2_S donors induces anti-inflammatory, antiapoptotic, and antioxidant activities, thereby reducing the toxicity of cisplatin.

**FIGURE 6:**
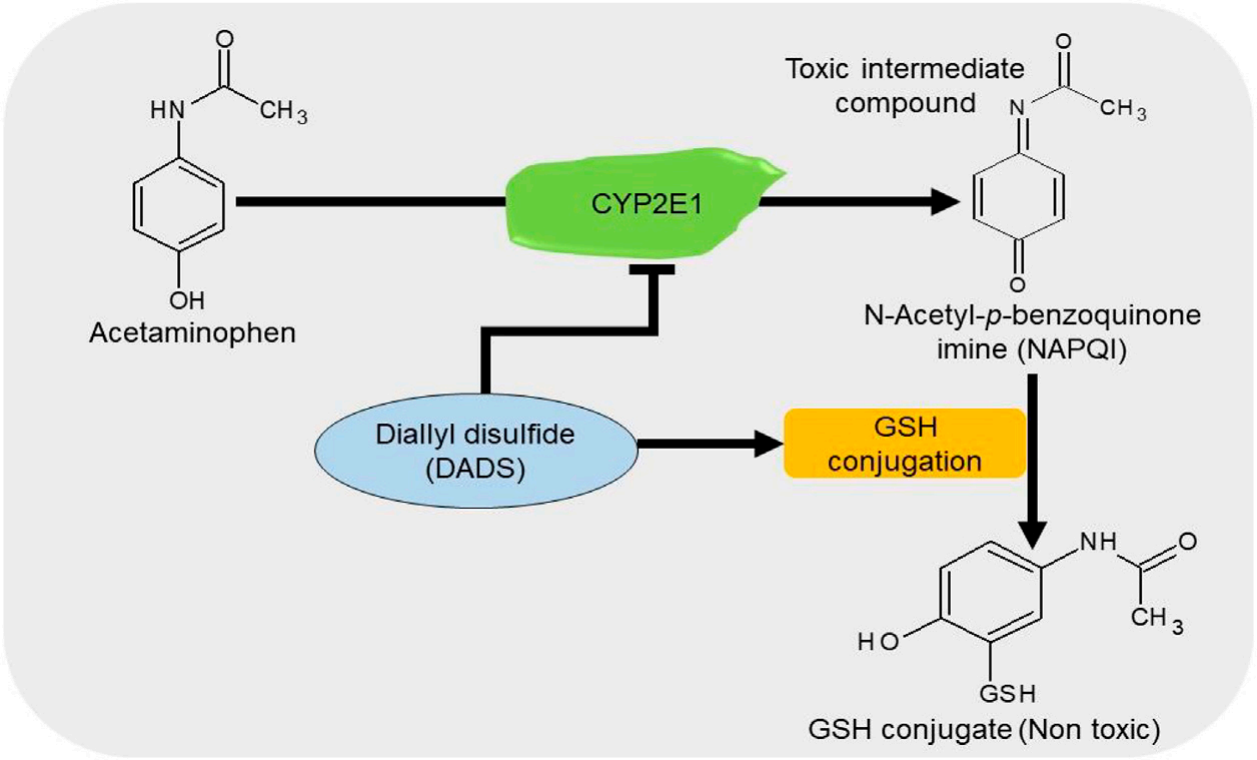
The diagrammatic presentation of the inhibitory effect of H_2_S donors on cytochrome p450 2E1 (CYP2E1). H_2_S donors increase the GSH levels and suppress the expression of CYP2E1, an enzyme responsible for the metabolism of APAP. By reducing the enzyme, H_2_S donors prevent the formation of a toxic intermediate metabolite NAPQI and by increasing GSH level, it facilitates the conjugation of NAPQI to form a nontoxic metabolite. H_2_S, hydrogen sulfide; CYP2E1, cytochrome p450 2E1; GSH, glutathione; APAP, acetaminophen; NAPQI, N-acetyl-p-benzoquinone imine.

APAP/paracetamol-induced nephrotoxicity is amongst the least causatives of renal injury (Hiragi et al., 2018). The overdose of paracetamol is associated with elevated serum levels of creatinine and urea, oxidative stress (Canayakin et al., 2016), apoptosis, and DNA damage (Lorz et al., 2004). Cytochrome P450 2E1 (CYP2E1) plays a vital role in the bioactivation of APAP (Lee et al., 1996). Treatment with DADS inhibits the APAP-induced increase in oxidative stress, apoptosis, and inflammation in rats by downregulating the expressions of NF-қB, TNF-α, and cyclooxygenase-2 (Cox-2) in the kidney and the level of CYP2E1 in the liver and kidney (Ko et al., 2017). It has been shown that treatment with 50 *μ*M/kg NaHS improves glomerular structures and functions by suppressing apoptosis and promoting anti-inflammatory and antioxidant activities in APAP-treated mice (Ozatik et al., 2019). These data suggest that H_2_S donors can prevent the bioactivation of APAP together with the associated apoptotic, inflammatory, and oxidative activities by regulating their respective pathways (Figure 6).

**FIGURE 7:**
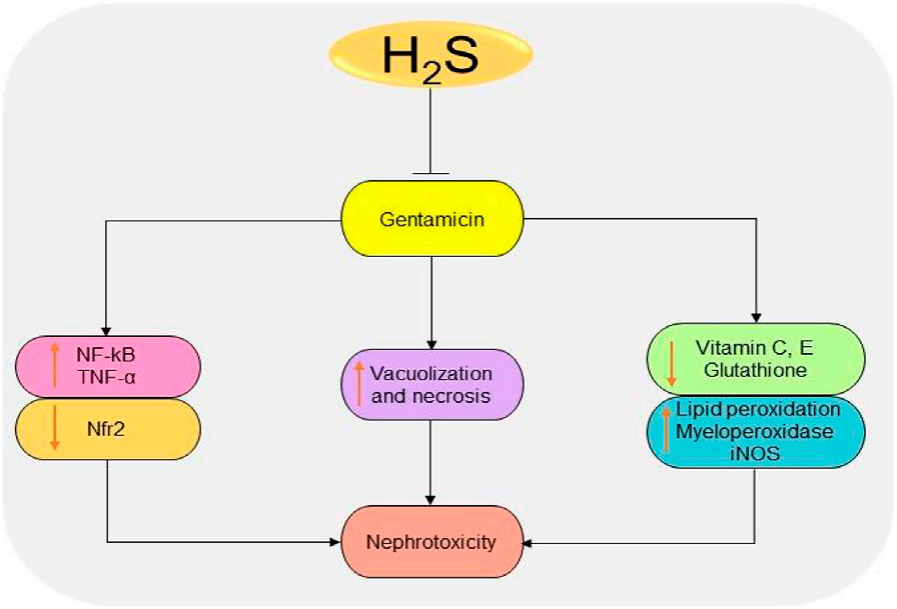
The diagrammatic illustration of the inhibitory effects of H_2_S donors on gentamicin-induced nephrotoxicity. H_2_S donors prevent gentamicin-induced oxidative stress, inflammation, and necrosis by reducing superoxide formation, lipid peroxidation, iNOS, NF-κB, TNF-α, and malondialdehyde levels. H_2_S, hydrogen sulfide; iNOS, inducible nitric oxide synthase; NF-κB, nuclear factor kappa B; TNF-α, tumor necrosis factor-alpha; ROS, reactive oxygen species; Nfr2, nuclear factor erythroid 2-related factor 2.

Gentamicin is a common antibiotic for the treatment of Gram-negative bacteria infections (Becker and Cooper, 2013). However, high risk of nephrotoxicity has been associated with the drug (Morin et al., 1980; Weir and Mazumdar, 1994). H_2_S protects the kidney from gentamicin-induced renal failure by regulating oxidative stress and inflammatory cytokines (Pedraza-Chaverrí et al., 2003; Pedraza-Chaverrí et al., 2003; Kalayarasan et al., 2009). It has been shown that treatment with NaHS could significantly reduce serum urea and creatinine levels, as well as improving glomerulus morphology in gentamicin-treated mice (Otunctemur et al., 2014). In gentamicin-treated rats, administration of 50 mg/kg/day DADS for 4 days induces renoprotective effects by promoting antioxidant and anti-inflammatory activities (Pedraza-Chaverrí et al., 2003). Correspondingly, treatment with 150 mg/kg/day diallyl sulfide for 6 days improves renal functions by downregulating NF-κB, inducible NO synthase (iNOS), and TNF-α and activating Nfr2 pathway (Kalayarasan et al., 2009). In summary, the antioxidant and anti-inflammatory effects observed following the inhibition or elevation of H_2_S levels play a key role in reducing the toxicity induced by gentamicin (Figure 7). However, more studies are needed to investigate the underlying mechanisms.

### Metal-Induced Nephrotoxicity

The accumulation of heavy metals such as lead, uranium (U), cadmium (Cd), mercury, zinc, and arsenic (As) has been suggested to induce toxicity in the kidney especially the nephron (Diamond and Zalups, 1998). H_2_S treatment attenuates U-induced ROS production, inflammation, ER stress, and caspase-dependent apoptosis (Yi et al., 2018). In the U-intoxicated rat model, NaHS alleviates the inflammatory and oxidative responses by inhibiting the activation of NF-қB and downstream levels of TNF-α, iNOS, and Cox-2 and promoting the activation of Nfr2 (Zheng et al., 2015). In addition, NaHS reduces the ER stress-mediated activation of apoptotic pathways by regulating Akt/glycogen synthase kinase-3β/Fyn/Nfr2 pathways in U-induced rats (Yi et al., 2018). Furthermore, diallyl trisulfide (DATS) can prevent As-induced elevation of apoptotic, inflammatory, and ROS activities in rats by activating Akt and Nfr2 antioxidant response element pathways (Miltonprabu et al., 2017). Similarly, diallyl tetrasulfide (DTS) significantly decreases the accumulation of Cd and the associated biochemical changes (Pari and Murugavel, 2005). The renoprotective effects of DTS are associated with metal chelating and antioxidant properties (Pari et al., 2007). Collectively, H_2_S donors can restore the reduced H_2_S levels and regulate key cellular activities altered by the toxicity of heavy metals.

### Calcium Oxalate Urolithiasis

Urolithiasis is the condition originated from the kidney, characterized by the accumulation of hard deposits of mineral crystals or salts usually made up of calcium salts, uric acids, and cysteine (Spivacow et al., 2016). Kidney stone results from the collection of calcium oxalate (CaOx), is termed calcium oxalate urolithiasis, and is one of the most common types of urolithiasis in human (Gisselman et al., 2009). Two forms of CaOx, namely, calcium oxalate monohydrate (COM) and calcium oxalate dihydrate (COD), are known to participate in body physiology and pathology, with COM being the nonpathogenic form and COD the pathogenic form (Manissorn et al., 2017). In addition, low pH favors COM formation, whereas the vice versa favors COD. A previous study shows that treatment with H_2_S donors could significantly reduce calcium oxalate stones agglomeration by adjusting the pH and facilitating calcium complex formations (Vaitheeswari et al., 2015). Treatment with 1.75 and 3.5 mM NaHS and Na_2_S_2_O_3_ could show an inhibition of 30–40% and 27–45%, respectively, following the analysis of calcium oxalate urolithiasis crystals from patients’ urine. In a CaOx rat model, administration of allicin, DADS, and DATS can prevent the accumulation of CaOx crystals and increase the expression of connexin 43 (Cx43) and the activities of gap junction, the effects which can be associated with CaOx stone progression (Lai et al., 2019). These data suggest that H_2_S donors possess potential in the treatment of kidney stones by regulating Cx43 level, pH, and the function of gap junction.

## Clinical Trials for Hydrogen Sulfide Donors in Common Kidney Diseases

Several clinical trials have been conducted to check the potential of H_2_S donors in patients. It has been demonstrated that treatment with H_2_S donor SG-1002 significantly increases the blood levels of H_2_S and brain natriuretic peptide and NO bioavailability in heart failure patients (Polhemus et al., 2015). Moreover, treatment with sulfhydryl-containing ACE inhibitor, zofenopril, induces vasculoprotective effects in essential hypertensive patients by reducing the oxidation of low-density lipids, atherosclerosis, and stabilizing NO signaling pathway (Napoli et al., 2004). In mild-to-moderate hypertension patients, the treatment with 30 mg/day zofenopril induces much reduction in BP and less severe adverse events compared to the treatment with 20 mg/day enalapril (Mallion, 2007). The drug also shows similar effects on other antihypertensive drugs such as candesartan (Leonetti et al., 2006), irbesartan (Malacco et al., 2015), and hydrochlorothiazide (Lacourciere and Provencher, 1989). Besides, the administration of the drug in combination with hydrochlorothiazide exhibits stronger effects in BP regulation compared to individual drugs alone (Malacco and Omboni, 2007). It has also been shown that tadalafil can effectively improve kidney functions and quality of life in patients with ESRD who undergoes hemodialysis (Bolat et al., 2017). Administration of ATB-346 in healthy individuals shows less adverse events and no hematological, cardiovascular, or renal effects in phase 1 clinical trials for the drug (Wallace, 2015). The drug is also confirmed in phase 2 clinical trial to induce anti-inflammatory responses and inhibits cyclooxygenase enzyme more efficiently than a nonsteroidal anti-inflammatory drug, naproxen with less gastrointestinal toxicity (Wallace et al., 2020). Despite the availability of a few clinical studies, H_2_S-containing drugs have indicated promising potential in the treatment of kidney diseases and the reduction of inflammatory responses.

## Conclusion and Future Direction

H_2_S is a ubiquitous gasotransmitter that plays an important role in cell signaling and is associated with the development and progression of numerous diseases. Several studies have revealed low levels of CBS, CSE, and 3-MPST in various renal diseases and demonstrated that H_2_S donor treatments can effectively improve the disease states. H_2_S donors generally restore normal renal functions by regulating inflammatory, apoptotic, oxidative stress, and autophagy pathways. Herein, we summarize the effects of H_2_S-releasing compounds on common renal diseases in animal models (Table 1). Improved therapeutic outcomes have been demonstrated in CKD. However, there is a deficiency of drug toxicity data on whether the treatment with H_2_S donors can impair kidney functions especially when they are used for a long time in treating CKD. The mechanisms of action of H_2_S donors in common renal diseases need to be further investigated. In addition, the respective effects of underproduction or overproduction as a result of the treatment with H_2_S inhibitors/donors need to be determined. Consistent with the summarized available data, we propose that reduced level of H_2_S in blood can be considered as a diagnostic tool or disease severity indicator for renal diseases after setting limit for H_2_S in normal and disease state. Regardless, considering that H_2_S is a promising option in treating normal renal diseases, further studies are needed to test the hypothesis at the clinical level. Furthermore, novel H_2_S donors can be designed and applied in the treatment of common renal diseases.

**TABLE 1:** Effects of H_2_S donors on common renal diseases in animal models.

Animals	Experimental models	Administered drugs	Effects	Proposed mechanisms	Ref
Mice	Lipopolysaccharide-induced AKI/sepsis-associated AKI	NaHS 50 *μ*mol/kg i.p. once for 6 h	Prevents inflammation and oxidative stress	Reduced expressions of TNF-α, IL-1β, MDA, MPO, H_2_O_2_, and caspase-1	(Chen Y. et al., 2018)
				Inhibition of TLR4/NLRP3 pathway	
Male Wister rats	5/6 nephrectomy CDK	NaHS 30 *μ*mol/kg twice daily for 8 weeks	Improves survival rate, body weight, oxidative stress, autophagy, inflammation, ECM remodeling, and apoptosis	Suppression of MDA/SOD properties and creatinine contents	(Askari et al., 2018; Shirazi et al., 2019)
				Decreased p-NF-κB and cleaved caspase 3 levels	
				Reduction of GSH-Px activities, Beclin, LC3A/B, IL-1β, TNF-α, MMPs, iNOS/eNOS status, VEGF, HIF1-α, and α-klotho	
				Deactivation of mTOR and activation of Nfr2 pathway	
	Adenine-induced CKD	i.p. NaHS 100 *μ*mol/kg/day for 4 weeks	Improves kidney function and renal injury; reduces cytotoxicity, ROS, and apoptosis	Reduction of TNF-α, IL-6, IL-10, NF-κB, MCP-1, MDA/SOD status, GSH-Px activities, p-MAPK, Bax, cleaved caspase-3, and Bcl-2 levels	(Wu et al., 2017)
Sprague-Dawley rats	STZ-induced obesity/obesity-associated kidney fibrosis	i.p. NaHS 100 *μ*mol/kg/day for 8 weeks	Reduces collagen deposition and inflammation	Reduction of kidney hydroxyproline contents	(Zeng et al., 2016; Li et al., 2017)
				Improved TIMPs/MMPs status	
				Suppression of Cx-40, Cx-43, Cx-45, MMP-2, MMP-7, MMP-8, MMP-11, and MMP-14	
				Inhibition of TGF-β1 and ERK1/2 pathways	
	Diabetes mellitus-induced renal fibrosis	i.p. NaHS 56 *μ*mol/kg/day for 4 weeks	Reduces collagen deposition, renal damage, and inflammation	Reduction of BUN, SCr, IL-1β, IL-6, TNF-α, and Col-IV levels	(Jia et al., 2018)
				Deactivation of TGF-β1/Smad3 pathway	
Mice	UUO-induced kidney fibrosis/injury	i.p. NaHS (1.12, 5.6, or 28 *μ*g/kg of BW) for 6 days after UUO induction	Reduces collagen deposition, oxidative stress, inflammation, and expansion of interstitium in the kidneys	Elevation of MnSOD, CuZnSOD, and catalase levels	(Jung et al., 2013)
				Suppression of TGF-β1/NF-κB and *p*-Smad3	
		i.p. NaHS 5.6 *μ*g/kg/day for 3 days before and 7–14 days after UUO induction	Reduces apoptosis, autophagy, and oxidative stress	Reduced expressions of Bax, cleaved caspase-3, cleaved caspase-9, cytochrome c, LC3-II, Beclin-1, Bcl-2, p-AMPK/AMPK, CAT, SOD, and p62	(Chen Q. et al., 2018)
		i.p. NaHS 50 *μ*g/kg/day	Reduces macrophage infiltration and inflammation	Reduction of TNF-α, IL-1β, IL-6, CD206, Arg-1, TIMP-1, NLRP3, NF-ΚB, and IL-4/STAT6 levels	(Zhou et al., 2020)
Sprague-Dawley rats		NaHS; 5.6, 56, and 560 *μ*g/kg/day for 7–14 days	Reduces inflammation, myofibroblast, and ECM deposition at 5.6 and 56 *μ*g/kg/day	Inhibition of MAPKs and TGF-β1 pathways	(Song et al., 2014)
Wister rats		NaHS 5 mg/kg/day for 3 days before UUO induction and 9 days after	Reduces macrophage accumulation, inflammation, and oxidative stress	Reduction of TNF-α expressions	(Jiang et al., 2014)
Lewis rats		i.p. 200 *μ*mol/kg GYY4137 for 30 days	Reduces cortical cortex, inflammation, apoptosis migration, and fibrosis	Suppression of serum creatinine and proteinuria, CD68, ANGIIR1, Col1α1, fibronectin, vimentin, TGF-β1, TGF-β1R2, E-cadherin, and Smad7 expressions	(Lin et al., 2018)
Mice	HHcy-induced-heterozygous CBS (CBS^+/−^) mice	NaHS, 30 µM in drinking water for 8 weeks	Reduces ECM accumulation, smooth muscle proliferation, and fibrosis	Reduction of Cx-40 and Cx-43 levels	(Pushpakumar et al., 2019)
Sprague-Dawley rats	STZ-induced diabetic kidney injury	i.p. S-propargyl-cysteine 65 mg/kg for 9 weeks	Improves renal function, inflammation, ECM accumulation, and oxidative stress	Suppression of IL-1β, MCP-1, TNF-α, and p-Stat3 levels	(Qian et al., 2016)
				Inhibition of TGF-β1, ERK, and p38 pathways	
White albino rats		i.p. NaHS 30 *μ*mol/kg/day and 100 µmol/kg/day for 30 days	Reduces oxidative stress, inflammation, and apoptosis	Reduction of caspase-3, p53, and SIRT1 activities	(Ahmed et al., 2019)

H_2_S, hydrogen sulfide; TNF-α, tumor necrosis factor-alpha; IL, interleukin; MDA, malondialdehyde; MPO, myeloperoxidase; H_2_O_2_, hydrogen peroxide; TRL4, Toll-like receptor 4; NLRP3, NOD-, LRR-, and pyrin domain-containing protein 3; SOD, superoxide dismutase; NF-κB, nuclear factor kappa B; GSH-Px, glutathione peroxidase; LC3A/B, microtubule-associated proteins 1A/B-light chain 3; MMPs, matrix metalloproteinases; eNOS, endothelial nitric oxide synthase; iNOS, inducible nitric oxide synthase; VEGF, vascular endothelial growth factor; HIF1α, hypoxia-inducible factor-1 alpha; mTOR, mammalian target of rapamycin; Nfr2, nuclear factor erythroid 2-related factor 2; MCP-1, monocyte chemoattractant protein-1; MAPK, mitogen-activated protein kinase; Bcl-2, B-cell lymphoma 2; Bax, Bcl-2-associated X protein; TIMP, tissue inhibitor of metalloproteinases; Cx, connexin; TGF-β1, transforming growth factor-beta 1; ERK1/2, extracellular signal-regulated protein kinases 1/2; BUN, blood urea nitrogen; Scr, serum creatinine; Col, collagen; Smad2/3, mothers against decapentaplegic homolog 2/3; AMPK, AMP-activated protein kinase; CAT, cysteine aminotransferase; P62, nucleoporin p62; COD206, cluster of differentiation 206; Arg-1, arginase-1; STAT, signal transducer and activator of transcription; CD68, cluster of differentiation 68; ANGIIR1, angiotensin II receptor 1; Col1α1, collagen type 1 alpha 1; TGF-β1R2, transforming growth factor-beta 1 receptor 2; p53, tumor.

## Author Contributions

Conceptualization: EN, MS, AA, DW, SD, XJ; data curation: EN, MS, AA, NK, SK, XZ, TL; funding acquisition: XJ, DW; writing-original draft: EN, NK, SK; visualization and supervision: MS, AA, SD, DW, XJ; writing-review and editing: EN, MS, AA, DW.

## Conflict of Interest

The authors declare that the research was conducted in the absence of any commercial or financial relationships that could be construed as a potential conflict of interest.
